# Conservation of molecular and cellular phenotypes of invariant NKT cells between humans and non-human primates

**DOI:** 10.1007/s00251-019-01118-9

**Published:** 2019-05-23

**Authors:** Krystle K. Q. Yu, Damien B. Wilburn, Joshua A. Hackney, Patricia A. Darrah, Kathryn E. Foulds, Charlotte A. James, Malisa T. Smith, Lichen Jing, Robert A. Seder, Mario Roederer, David M. Koelle, Willie J. Swanson, Chetan Seshadri

**Affiliations:** 1 Department of Medicine, University of Washington, Seattle, WA USA; 2 Department of Genome Sciences, University of Washington, Seattle, WA USA; 3 Vaccine Research Center, National Institute of Allergy and Infectious Diseases, National Institutes of Health, Bethesda, MD USA; 4 Department of Pathology, Molecular Medicine and Mechanisms of Disease Program, University of Washington, Seattle, WA USA; 5 Department of Laboratory Medicine, University of Washington, Seattle, WA USA; 6 Department of Global Health, University of Washington, Seattle, WA USA; 7 Benaroya Research Institute, Seattle, WA USA; 8 Vaccine and Infectious Diseases Division, Fred Hutchinson Cancer Research Center, Seattle, WA USA; 9 Tuberculosis Research & Training Center, University of Washington, Seattle, WA USA

**Keywords:** CD1D, iNKT cells, Non-human primate, T cell receptor

## Abstract

Invariant NKT (iNKT) cells in both humans and non-human primates are activated by the glycolipid antigen, α-galactosylceramide (α-GalCer). However, the extent to which the molecular mechanisms of antigen recognition and in vivo phenotypes of iNKT cells are conserved among primate species has not been determined. Using an evolutionary genetic approach, we found a lack of diversifying selection in CD1 genes over 45 million years of evolution, which stands in stark contrast to the history of the MHC system for presenting peptide antigens to T cells. The invariant T cell receptor (TCR)-α chain was strictly conserved across all seven primate clades. Invariant NKT cells from rhesus macaques (*Macaca mulatta*) bind human CD1D-α-GalCer tetramer and are activated by α-GalCer-loaded human CD1D transfectants. The dominant TCR-β chain cloned from a rhesus-derived iNKT cell line is nearly identical to that found in the human iNKT TCR, and transduction of the rhesus iNKT TCR into human Jurkat cells show that it is sufficient for binding human CD1D-α-GalCer tetramer. Finally, we used a 20-color flow cytometry panel to probe tissue phenotypes of iNKT cells in a cohort of rhesus macaques. We discovered several tissue-resident iNKT populations that have not been previously described in non-human primates but are known in humans, such as TCR-γδ iNKTs. These data reveal a diversity of iNKT cell phenotypes despite convergent evolution of the genes required for lipid antigen presentation and recognition in humans and non-human primates.

**Electronic supplementary material:**

The online version of this article (10.1007/s00251-019-01118-9) contains supplementary material, which is available to authorized users.

## Introduction

T cells are activated by antigens bound to antigen-presenting molecules on the surface of antigen-presenting cells through interactions with cognate T cell receptors (TCRs). The most widely studied antigen presenting systems are the major histocompatibility complex (MHC) class I and II genes which facilitate the presentation of peptide antigens to T cells (Garcia et al. 1996). Humans express three MHC class I genes, designated HLA-A, HLA-B, and HLA-C, which are among the most polymorphic in the human genome. It is a selective advantage not only for an individual to express several class I molecules to bind different sets of peptide antigens but for a population to have many alleles segregating among its members (Carrington et al. 1999; Wills and Green 1995). Orthologs of human HLA-A, HLA-B, and HLA-C have only been identified in African apes (chimpanzee, bonobo, gorilla, and orangutan) but not in Old World monkeys (macaques and baboons) (Adams and Parham 2001), all of which are considered simian primates. Other primate species have either homologous loci or deletions and duplications of these genes. Thus, the evolution of MHC class I genes in primates reflects the selective adaptation to the unique immune pressures of each species.

The CD1 family of antigen-presenting molecules is highly similar to MHC class I genes except that it evolved to present lipid rather than peptide antigens to T cells. Humans express five CD1 genes (CD1A, CD1B, CD1C, CD1D, and CD1E) that vary in the types of lipids that they bind, as well as their patterns of cellular expression and intracellular trafficking (Kasmar et al. 2009). The five human CD1 genes are tightly clustered on chromosome 1, while the MHC loci are separately located on chromosome 6. In the chicken genome, two CD1 genes are syntenic with the MHC locus, suggesting that CD1 was present in an ancient version of the MHC locus that arose at least 300 million years ago at the time of the last common ancestor of avians and mammals (Salomonsen et al. 2005). Subsequently, the CD1 and MHC loci separated as a result of recombination to a different chromosome.

CD1 gene families have been identified in nearly every mammalian genome that has been sequenced to date (Reinink and Van Rhijn 2016). The most extensively studied is the murine system which contains only one ortholog of human CD1D (Balk et al. 1991). Both human and mouse CD1D activate a specialized population of T cells called invariant NKT (iNKT) cells. Murine iNKT cells are able to lyse human CD1D-expressing target cells pulsed with α-galactosylceramide (α-GalCer) (Ishihara et al. 2000; Nicol et al. 2000; Nieda et al. 1999). Human CD1D tetramers loaded with α-GalCer are able to bind mouse iNKT cells defined by NK1.1 expression (Karadimitris et al. 2001). Crystal structures of human and mouse CD1D have been solved and show that the molecular interactions between CD1D and the iNKT cell TCR are highly similar (Borg et al. 2007). Further, human iNKT cell TCRs bind to mouse CD1D loaded with α-GalCer with an affinity that is comparable to the mouse iNKT cell TCR (Kjer-Nielsen et al. 2006). Collectively, these data suggest cross-species conservation of the molecular mechanism of antigen recognition by iNKT cells. However, there are also notable differences between human and mouse iNKT cells. Human iNKT cells express a semi-invariant TCR consisting of a germline rearrangement of TRAV10 and TRAJ18 in the TCR-α chain and a limited diversity of TCR-β chains, the most common of which is TRBV25 (Dellabona et al. 1994; Porcelli et al. 1993). The mouse iNKT cell TCR is highly similar but not identical. Of the 25 residues within the TCR-α shown to mediate contact with CD1D or α-GalCer, eight are not conserved between mouse and human (Borg et al. 2007). There is even less conservation within the TCR-β with different gene segment usage and none of the seven residues showing conservation between species (Borg et al. 2007). These data show that while CD1D and iNKT cells have remained associated through evolution, the molecular mechanisms facilitating their interactions may differ between species.

A number of studies have begun to elucidate the functions of iNKT cells in non-human primates. Murine CD1D tetramers loaded with α-GalCer with or without monoclonal antibodies targeting specific epitopes on the invariant TCR-α chain have been used to isolate iNKT cells from rhesus (*Macaca mulatta*), cynomolgus (*Macaca fascicularis*), and pig-tailed (*Macaca nemestrina*) macaques (Fernandez et al. 2012, 2009; Gansuvd et al. 2008; Liu et al. 2006; Motsinger et al. 2003). More recently, human CD1D tetramers loaded with α-GalCer were used to study iNKT cells in sooty mangabeys (*Cercocebus atys*) (Rout et al. 2012, 2010a, b). Experiments conducted with α-GalCer pulsed human dendritic cells and human or mouse CD1D-transfected cell lines confirmed CD1D-dependent antigen recognition and activation of iNKT cells in non-human primates (Motsinger et al. 2003; Rout et al. 2010b). Similar to mouse and human iNKT cells, iNKT cells present in the peripheral blood of non-human primates produce large amounts of IFN-γ and IL-4 upon stimulation with α-GalCer and display an effector memory phenotype characterized by expression of CD45RO, CCR7, and CD28 but not CD62L (Gansuvd et al. 2003; Rout et al. 2010b). Despite these similarities, a number of notable differences have also been observed. In humans, canonical iNKT cells express the TRBV25 gene segment, while in cynomolgus and rhesus macaques, this does not seem to be the case (Gansuvd et al. 2003; Motsinger et al. 2003). Human blood iNKT cells are typically CD4^+^ or CD4^−^CD8^−^ and express CD161 while blood iNKT cells in rhesus macaques are usually CD8^+^ and lack CD161 (Gansuvd et al. 2003). Finally, there is very little data regarding tissue-specific phenotypes of iNKT cells in non-human primates.

We addressed how CD1 genes and iNKT cells are conserved among humans and non-human primates using evolutionary genetics as well as molecular and cellular immunology approaches. In stark contrast to the MHC system for peptide antigen presentation, we find that the five-gene CD1 locus for lipid antigen presentation has remained intact without significant duplications or deletions over approximately 45 million years of simian evolution. Gene segments encoding the semi-invariant TCR-α were conserved across all primate clades, revealing a remarkable example of convergent evolution between genes encoding antigen presentation and recognition. Cloning and reconstitution studies confirmed that the rhesus iNKT cell TCR was sufficient to bind human CD1D tetramer and become activated by human CD1D transfectants. Finally, we used multi-parameter flow cytometry to discover tissue-specific iNKT cell populations in non-human primates that have human analogs. Thus, iNKT cells exist as diverse cellular phenotypes in both humans and non-human primates despite strict conservation of the genes mediating lipid antigen presentation and recognition.

## Materials and methods

### Evolutionary genetics

Primate CD1 homolog sequences were acquired using a combination of existing repositories. For human, chimpanzee, gorilla, orangutan, gibbon, olive baboon, and marmoset, homolog sequences were downloaded from Ensembl version 89, and only homologs located on chromosome assemblies were included. For green monkey and crab-eating macaque, custom scripts were used to reconstruct likely open reading frames (ORFs) from available reference genomes. Briefly, the human CD1 homolog sequences were used as BLAST queries to identify putative exons, gene coordinates were identified based on exon contiguity within 20 kb, and likely open reading frames were extracted from these loci using Exonerate version 2.2 with human CD1 sequences as queries. Rhesus macaque CD1 cDNA sequences were retrieved from Genbank (accession #AB458511-AB458513, NM001033114, AY094979). A CD1 gene tree was constructed from the primate CD1 sequences, in addition to the two chicken homologs available in Ensembl for rooting, by first aligning sequences using Fast Statistical Alignment (FSA) version 1.15.9, then constructing a maximum likelihood tree using Randomized Axelerated Maximum Likelihood (RaxML) version 8.2.8 with the PROTGAMMAJTT model and bootstrap probabilities calculated from 200 replicates.

Following identification as TRAV10 and TRAJ18 in rhesus macaques, the human sequences for each locus were used as queries in nucleotide BLAST searches to identify likely orthologous regions in additional simian genomes (chimpanzee, gorilla, orangutan, gibbon, rhesus macaque, crab-eating macaque, baboon, green monkey, marmoset). The identified locus with the lowest *e* value was extracted using the “faidx” command in SAMtools, translated in the correct reading frame, and aligned using FSA.

### Animals

Peripheral blood mononuclear cells (PBMC) from a healthy 3-year-old female Chinese rhesus macaque were isolated from whole blood by a standard Ficoll method and cryopreserved in liquid nitrogen. The cells were used to obtain data in Figs. 2 and 3. Samples used for tissue staining in Fig. 4 were from cryopreserved tissues from 9- to 11-year-old male Indian-origin rhesus macaques that had previously undergone necropsy following various malaria challenges or BCG vaccination protocols (Online Resource 1).

Livers were processed as previously described (Epstein et al. 2011). Spleen and lymph nodes were dissociated by passing tissue through a 70-μm cell strainer using a 6-mL syringe plunger. Splenocytes were further separated by Ficoll gradient centrifugation. Lung tissue was processed in a GentleMacs Dissociator (Miltenyi Biotec, Bergisch Gladbach, Germany) according to manufacturer’s instructions including enzymatic digestion (collagenase I/DNAse) for 30 min in 37 °C with shaking at 200 rpm. All cells were cryopreserved in 10% DMSO/FCS and stored in liquid nitrogen until use.

### Generation of human CD1D tetramers

PBS-57 (α-galactosylceramide or α-GalCer)-loaded and unloaded human CD1D monomers were provided by the National Institutes of Health Tetramer Core Facility (Emory University, Atlanta, GA). Tetramers were prepared as previously described (Liu et al. 2006). Briefly, 10 μL of the loaded or unloaded stock monomers was incubated with 19 μL of streptavidin conjugated to APC (Life Technologies, Carlsbad, CA), PE (Life Technologies, Carlsbad, CA), DyLight 488 (Thermo Fisher Scientific, Rockford, IL), or BV650 (BioLegend, San Diego, CA) that were titrated in at ten aliquots of 1.9 μL every 10 min to facilitate tetramerization. The tetramer was filtered through a SpinX column (Sigma, St. Louis, MO) to remove aggregates and stored at 4 °C until use.

### Culture media

Media (R10) for washing PBMC consisted of RPMI 1640 (Gibco, Waltham, MA) supplemented with 10% fetal bovine serum (FBS) (Hyclone, Logan, UT). Our base T cell media (TCM) consisted of RPMI 1640 supplemented with 10% FBS, 100 U/mL penicillin, 100 mg/mL streptomycin, 55 mM 2-mercaptoethanol, 0.3X essential amino acids, 60 mM non-essential amino acids, 11 mM HEPES, and 800 mM L-glutamine (Gibco, Waltham, MA) sterile-filtered. Our TCM containing human serum (TCM/HS) consisted of 10% human serum (derived from healthy donors), 100 U/mL penicillin, 100 mg/mL streptomycin, and 400 mM L-glutamine (Gibco, Waltham, MA). Enhanced RPMI for Jurkat cell culturing consisted of RPMI 1640 (Gibco, Waltham, MA) supplemented with 10% FBS (Hyclone, Logan, UT), 800 mM L-glutamine (Gibco, Waltham, MA), 100 U/mL penicillin, and 100 mg/mL streptomycin (Gibco, Waltham, MA).

### Tetramer staining

Rhesus PBMC were thawed in warm thaw media (R10 with 2 μL/mL Benzonase (Millipore, Billerica, MA) sterile-filtered) and centrifuged at 1500 rpm for 5 min. The supernatant was decanted, and the viable cells were enumerated by trypan blue (Millipore, Billerica, MA) exclusion. The cells were centrifuged at 1500 rpm for 5 min and rested overnight at a density of 2 million cells/mL. The following day, the cells were counted and plated at a density of 1 million cells/well in a 96-well U-bottom plate. The cells were washed with FACS buffer (1× phosphate-buffered saline (PBS) (Gibco, Waltham, MA) supplemented with 0.2% bovine serum albumin (BSA) (Sigma, St. Louis, MO)) and centrifuged at 1800 rpm for 3 min. They were then blocked with human serum (Valley Biomedical, Winchester, VA) and FACS buffer mixed 1:1 for 10 min at 4 °C. The wells were washed twice with FACS buffer and then resuspended in 50 μL FACS buffer with 4 μL of either unloaded CD1D tetramer or α-GalCer-loaded CD1D tetramer and incubated at room temperature for 40 min in the dark. At the end of the incubation period, the cells were washed twice with PBS and stained with Aqua Live/Dead stain (Life Technologies, Carlsbad, CA) according to the manufacturer’s instructions. Following a 15-min incubation at room temperature, the cells were washed twice in PBS and then labeled with anti-CD3 PerCP Cy5.5 (clone SP34-2) (BD Biosciences, San Jose, CA), anti-CD4 APC H7 (clone L200) (BD Biosciences, San Jose, CA), and anti-CD8α BV650 (clone RPA-T8) (BioLegend, San Diego, CA) antibodies for 30 min at 4 °C. After two final washes in FACS buffer, the cells were fixed in 1% paraformaldehyde (PFA) (Electron Microscopy Sciences, Hatfield, PA) and acquired on a BD LSRFortessa (BD Biosciences, San Jose, CA) equipped with blue (488 nm), green (532 nm), red (628 nm), violet (405 nm), and ultraviolet (355 nm) lasers.

### T cell sorting and expansion

Rhesus macaque PBMC were thawed and rested as described above and counted using trypan blue. The following day, the cells were stained with tetramer as described in “Materials and methods” but were resuspended in TCM rather than fixed with 1% PFA. The cells were filtered through a cell strainer tube (Falcon, Tewksbury, MA) prior to sorting to remove aggregates, and tetramer-positive T cells were collected at the UW Department of Immunology Flow Cytometry Core using a FACS Aria (BD Biosciences, San Jose, CA) cell sorter equipped with violet (407 nm), blue (488 nm), and red (641 nm) lasers.

Sorted T cells were washed and resuspended in TCM/HS. They were then divided between eight wells of a 96-well U-bottom tissue culture plate and seeded with 150,000 cells per well of irradiated human PBMC as feeder cells. Phytohemagglutinin (PHA) (Remel, San Diego, CA) was added to a final concentration of 1.6 mg/mL. After 2 days in culture at 37 °C/5% CO_2_, 10 μL human natural IL-2 (hnIL-2) (Hemagen, Columbia, MD) was added to each well. Half the media was replaced every 2 days with TCM/HS supplemented with 1:20 hnIL-2. When the cell clusters were large and round (approximately after 8 days of growth), they were pooled into a 24-well plate. After 10 days in culture, cell lines were screened by CD1D-α-GalCer tetramer staining as described above. Further expansion of the T cell line was performed using a modified version of a previously established rapid expansion protocol (Riddell et al. 1992). Briefly, 100,000 T cells were mixed with 5 million irradiated EBV-transformed B cells and 25 million irradiated PBMC as feeder cells in R10 in T25 tissue culture flasks (Costar, St. Louis, MO) with 25 mL TCM. Anti-CD3 (clone OKT3) was added at a final concentration of 30 ng/mL, and the mixture was incubated overnight at 37 °C/5% CO_2_. The following day, recombinant human IL-2 (rhIL-2) (Prometheus Pharmaceuticals through UWMC Clinical Pharmacy) was added at a final concentration of 50 U/mL. On day 4, the cells were washed twice in TCM to remove the anti-CD3 antibody and resuspended in fresh media supplemented with rhIL-2 at 50 U/mL. Half the media was replaced every 3 days or split into new T25 tissue culture flasks as determined by cell confluency. After 13 days in culture, the line was screened by tetramer staining and then cryopreserved on day 14.

### Intracellular cytokine staining

T cell lines were thawed and rested as described above. In parallel, 250,000 K562 antigen-presenting cells stably transfected with human CD1D (courtesy of D. Branch Moody, Brigham and Women’s Hospital) were loaded with 5 μg/mL α-GalCer (Avanti Polar Lipids, Alabaster, Alabama) per well for 18 h at 37 °C/5% CO_2_. The next day, the cells were centrifuged at 1800 rpm for 3 min, and 1 million cells per well were plated into a 96-well U-bottom plate. Stimulation was performed at 37 °C/5% CO_2_ for 6 h in the presence of 200,000 α-GalCer-loaded or control K562-CD1D cells and 10 μg/mL Brefeldin A (Sigma, St. Louis, MO). At the end of the incubation period, EDTA (Thermo Fisher Scientific, Waltham, MA) at a final concentration of 2 mM was added, and the plates were stored overnight at 4 °C. The following day, the samples were washed twice in PBS and then stained with Aqua Live/Dead (Life Technologies, Carlsbad, CA) prepared according to manufacturer’s instructions and incubated for 20 min at room temperature. Live/Dead staining and all steps following were performed in the dark. The cells were washed twice in PBS and then incubated at room temperature for 10 min in 1× FACS Lyse (BD Biosciences, San Jose, CA). Following one wash with FACS buffer, the cells were incubated an additional 10 min in 1× FACS Perm II (BD Biosciences, San Jose, CA) at room temperature. The cells were washed twice in FACS buffer and stained with markers for CD3, CD4, CD8α, IFN-γ (clone B27) (BD Biosciences, San Jose, CA), and TNF (clone Mab11) (eBioSciences, Waltham, MA) for 30 min at 4 °C. Following two final washes in FACS buffer, the cells were fixed in 1% PFA and acquired on a BD LSRFortessa (BD Biosciences, San Jose, CA).

### T cell receptor cloning and sequencing

RNA was extracted from rhesus-derived iNKT cell lines using a RNeasy Mini Kit (Qiagen, Hilden, Germany). cDNA was generated from 1 μg RNA using the SMARTer RACE 5′/3′ Kit (Clontech Laboratories, Inc., Mountain View, CA) and the manufacturer’s protocol for preparing 5′-RACE-Ready cDNA.

To determine the sequence of the rhesus T cell receptor (TCR)-β chain, 5′-RACE was performed according to the manufacturer’s protocol using the following gene-specific primer for the human TCR-β chain constant region: 5′-GATTACGCCAAGCTTCCCATTCACCCACCAGCTCAGCTCCACG-3′. The RACE products were resolved through a 1% agarose (Thermo Fisher Scientific, Waltham, MA) gel supplemented with 1× SYBR Safe (Life Technologies, Carlsbad, CA) for 1 h at 120 V, and the band located at ~ 700 bp was excised. The NucleoSpin Gel and PCR Clean-up kit (Macherey-Nagel, Duren, Germany) was used to extract DNA, and then In-Fusion Cloning of RACE Products was performed according to the 5′-RACE protocol according to manufacturer’s instructions. Plasmid DNA was extracted using the Plasmid Mini Kit (Qiagen, Hilden, Germany) and was submitted for sequencing (Genewiz, South Plainfield, NJ).

To determine the sequence of the rhesus TCR-α chain, primers for the human iNKT TCR-α chain constant region (5′-GATTACGCCAAGCTTGTTGCTCCAGGCCACAGCACTGTTGCTC-3′) and for rhesus Vα24 (TRAV10) (5′-ATGAAGAAGCGTCTGAGGACC-3′) were used. PCR was performed using Q5 High-Fidelity DNA Polymerase (New England BioLabs, Ipswich, MA) according to the manufacturer’s instructions. The PCR product was resolved through a 1% agarose gel supplemented with 1× SYBR Safe, and the band located at ~ 600 bp was excised. DNA was extracted using the NucleoSpin Gel and PCR Clean-up kit and sequenced. The sequencing results for both TCR-α and TCR-β were cleaned using Sequencher software version 5.2.4 and analyzed using the IMGT/V-QUEST program version 3.4.7. Sequences were aligned using Serial Cloner version 2.6.

### Modeling of the rhesus NKT and CD1D

Rhesus macaque CD1D-NKT interactions were modeled based on the crystal structure of the human orthologs (PDB 2po6). Using open-source PyMOL (The PyMOL Molecular Graphics System, version 1.8.61 Schrödinger, LLC), residues with nonsynonymous substitutions were mutated in silico with the lowest energy rotamer. Residues were subsequently colored based on predicted interactions with CD1D (blue), nonsynonymous mutations with conserved biochemical properties (green), and nonsynonymous mutations with differing biochemical properties (red).

### T cell receptor reconstitution

#### T cell receptor cassette construction

Codon-optimized rhesus-derived iNKT cell TCR sequences were assembled into a TCR cassette (Linnemann et al. 2013). In this construct, the TCR-α and TCR-β chain constant regions are replaced with modified murine TCR constant regions to facilitate measurement of TCR expression and to encourage pairing between exogenous TCR chains. The TCR cassettes were synthesized through Thermo Fisher GeneArt Synthesis service and were then cloned into pRRL.PPT.MP.GFPpre (Jing et al. 2016; Zhou et al. 2012) using *Bam*HI and *Sal*I restriction enzymes (New England BioLabs, Ipswich, MA). All plasmids were purified using Maxi Prep kits (Qiagen, Hilden, Germany).

#### Generation of lentivirus

Lenti-X HEK293T cells (Clontech, Mountain View, CA) were seeded at 2 million cells per 100 mm tissue culture dish and incubated for 48 h at 37 °C/5% CO_2_ in DMEM (Gibco, Waltham, MA) or until cells reached 75% confluency. The medium was replaced 4 h before transfection. Cells were transfected with 10 μg pRRL-TCR plasmid, 5 μg pCI-VSVG envelope plasmid, and 5 μg of a psPAX2 packaging vector (gifted from Dr. Stanley Riddell at Fred Hutchinson Cancer Research Center). Plasmids were mixed with Fugene 6 transfection reagent (Promega, Madison, WI) at a dilution of 1:12 in a total volume of 600 μL. Transfection mixture was added dropwise into the cell culture and incubated overnight in the conditions described above. The medium was then replaced and incubated for an additional 48 h. After this time, 20 μL of supernatant was titered using Lenti-X GoStix (Clontech, Mountain View, CA) per the manufacturer. Supernatant was then harvested every 12 h for a total of three collections. At each collection, cell debris was removed by centrifugation at 1500 rpm for 5 min, and cleaned supernatant was reserved in a 50-mL conical and kept at 4 °C until three collections had been acquired. Supernatant was then incubated overnight with Lenti-X concentrator (Clontech, Mountain View, CA) at a ratio of 1:3. The following day, the supernatant was centrifuged at 1500×*g* for 45 min at 4 °C. Supernatant was then discarded, and the pelleted virions were resuspended in 300 μL R10 media and stored at − 80 °C until further use.

#### Transduction of Jurkat cells

CD8-expressing Jurkat cells were seeded at 1 million cells per well in a 48-well plate in enhanced RPMI. The same day, the Jurkat cells were transduced with TCR lentivirus at an estimated multiplicity of infection (MOI) of 5 with 1 μL of polybrene at a final concentration of 4 μg/mL (Sigma, St. Louis, MO). Cells and virus were incubated for 4 h at 37 °C/5% CO_2_ and washed with PBS (Gibco, Waltham, MA) to remove excess virus. Jurkat cells were maintained in culture for 1 week using enhanced RPMI and screened for TCR expression (Jing et al. 2016) using an anti-mouse TCR-β chain APC antibody (BD Biosciences, San Jose, CA) and tetramer staining as described above.

### Multiparameter flow cytometry

To determine the phenotypes of iNKT cells present in blood and tissue, the following samples were analyzed in two batches: PBMC (*n* = 12), lung (*n* = 5), lymph node (*n* = 9), spleen (*n* = 9), and liver (*n* = 4) (Online Resource 1). Cryopreserved cells were thawed in warm (37 °C) R10 medium and centrifuged at 1500 rpm for 7 min. Between 1 and 5 million cells per well were plated in a 96-well V-bottom plate and were washed twice with FACS buffer and centrifuged at 2000 rpm for 3 min. They were then resuspended in 50 μL tetramer staining buffer with a pre-titrated amount of PBS-57 (α-GalCer)-loaded CD1D tetramer (NIH Tetramer Core Facility, Emory University, Atlanta, GA) and incubated at room temperature for 20 min protected from light. Cells were washed three times with PBS and stained with Ultraviolet Live/Dead stain (Life Technologies, Carlsbad, CA) for 20 min at room temperature. Cells were washed twice in FACS buffer and then immunostained with a cocktail of cell surface antibodies (Online Resource 2) for 20 min at room temperature. Following three washes with surface staining buffer, cells were fixed in 1% PFA (Electron Microscopy Sciences, Hatfield, PA) and acquired on a BD FACSymphony X-50 (BD Biosciences, San Jose, CA) equipped with blue (488 nm), green (532 nm), red (628 nm), violet (405 nm), and ultraviolet (355 nm) lasers.

Analysis was performed in FlowJo version 9.9.6. For Fig. 4b, the R openCyto “mindensity” function was used to gate all populations after the viability gate (e.g., Live > CD3^+^ > Tet^+^) (Finak et al. 2014).

## Results

### A five-gene CD1 locus is evolutionarily conserved among primates

**Fig. 1:**
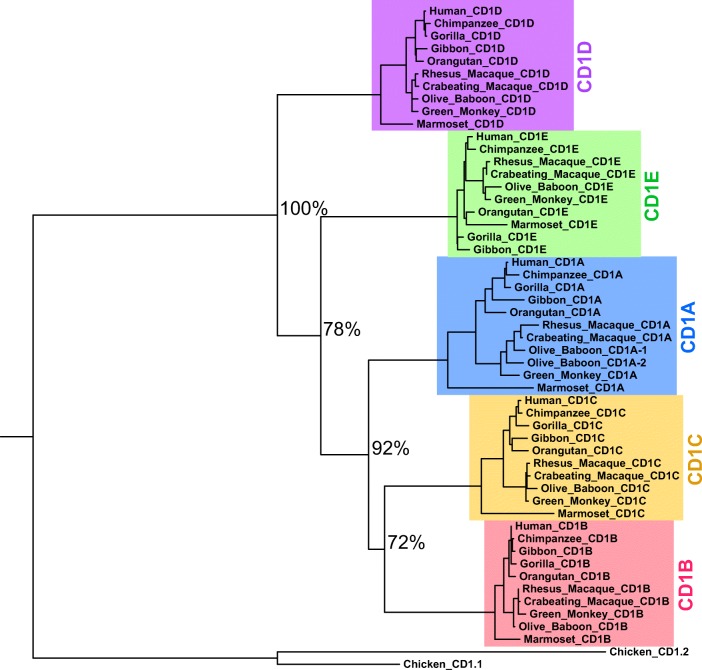
Conservation of the CD1 primate locus. Primate CD1 homolog sequences were acquired from a combination of bioinformatic sources. A maximum likelihood CD1 gene tree was constructed after including two chicken homologs for rooting. Apart from CD1A in olive baboon, each paralog exists as a single gene copy in each primate species and forms a phylogenetically distinct group that generally matches the species phylogeny (bootstrap probabilities are reported as percentages for separation of each paralog group). CD1D is likely the oldest gene and shows the most conservation among primate species

To explore the evolution of CD1 genes in primates, we constructed a gene tree of simian primate CD1 sequences, rooted against the chicken CD1 locus (Fig. 1). Remarkably, except for CD1A in olive baboon, each paralog exists as a single-gene copy in each primate species and forms a phylogenetically distinct group that generally matches the species phylogeny. From both these results and further examination of additional mammalian CD1 sequences (data not shown), CD1D appears to be the ancestral CD1 paralog. Through additional gene duplication events, CD1E likely arose next, followed by the other “Group 1” CD1 genes (CD1A, CD1B, and CD1C). These data broadly reveal that the CD1 gene cluster is highly conserved over 45 million years of primate evolution, which stands in stark contrast to the evolution of MHC class I over this same time period.

### Human CD1D tetramers identify iNKT cells in rhesus macaques

**Fig. 2:**
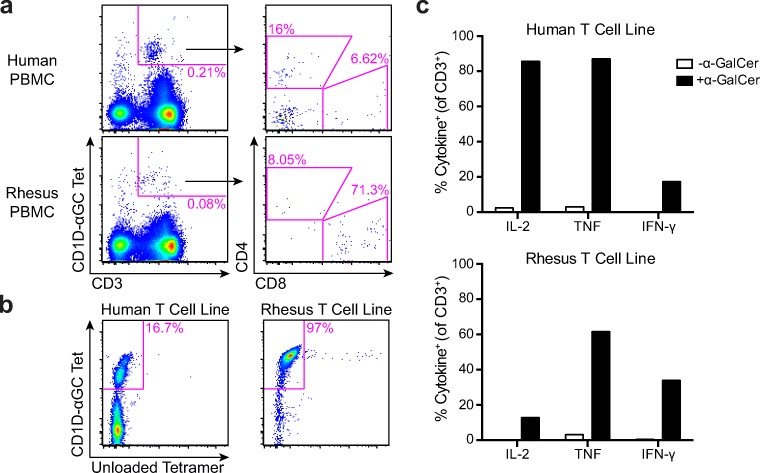
Rhesus iNKT cells stain with human α-GalCer-loaded CD1D tetramers and are activated by human CD1D-transfected antigen-presenting cells. **a** The frequency of α-GalCer-specific T cells was identified as CD3^+^ and CD1D-α-GalCer tetramer positive (CD1D-ɑGC Tet^+^). Shown is representative staining from a rhesus macaque (bottom) and human PBMC (top). Tetramer-positive cells were further examined for expression of CD4 or CD8α co-receptor. **b** CD1D-ɑGC Tet^+^ cells were sorted and expanded in vitro after which the T cell lines were screened with tetramer to determine specificity for α-GalCer. **c** Human K562 cells that were stably transfected with human CD1D were co-incubated with iNKT cells in the presence or absence of α-GalCer. Intracellular cytokine analysis shows that cells produce IL-2, TNF, and IFN-γ in the presence of human CD1D and α-GalCer. Data are expressed as percent cytokine positive of CD3^+^ cells. Sorting and T cell line data are representative of one rhesus macaque, but tetramer staining of T cells and the iNKT cell line was confirmed in at least two experiments. Intracellular cytokine staining was performed twice on the iNKT cell line, and a representative graph is shown

This genetic analysis and published studies demonstrating the use of human CD1D tetramers in sooty mangabeys led us to hypothesize that we could use human CD1D tetramers to isolate invariant NKT cells from rhesus macaques (Rout et al. 2012, 2010b). We used human CD1D tetramers loaded with α-GalCer to stain peripheral blood mononuclear cells from a healthy rhesus macaque and noted a distinct population of T cells bound to tetramer with a fluorescence intensity similar to that seen in humans (Fig. 2a). We further noted that tetramer-bound cells primarily expressed CD8α in rhesus macaques but were CD4^+^ and CD4^−^CD8^−^ in humans (Fig. 2a). These data extend previous studies in rhesus macaques using TCR-α-specific monoclonal antibodies (Gansuvd et al. 2008, 2003). To confirm the specificity of this staining, we sorted the tetramer-positive cells and expanded them in vitro for 4 weeks. The resulting T cell line consisted of 97% tetramer-bound cells with staining intensity similar to that observed in a human T cell line (Fig. 2b). We next sought to investigate whether rhesus-derived iNKT cells could generate a functional response to antigen presented by human CD1D. We co-incubated iNKT cells with K562 antigen-presenting cells stably transfected with human CD1D and pre-loaded with α-GalCer. Rhesus-derived iNKT cells produced IL-2, IFN-γ, and TNF only in the presence of α-GalCer and CD1D (Fig. 2c). Taken together, these data confirm and significantly extend previous studies by revealing that iNKT cells in rhesus macaques can be isolated using human CD1D tetramers and are activated by human CD1D transfectants.

### Human and non-human primate iNKT cell T cell receptor sequences are highly conserved

**Fig. 3:**
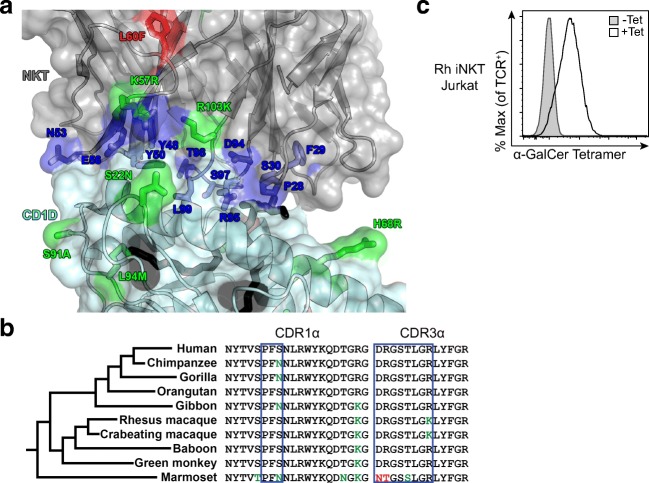
The human and non-human primate iNKT cell TCR is highly conserved. **a** Modeling of rhesus macaque CD1D-NKT interactions based on the crystal structure of the human orthologs (PDB 2po6). α-GalCer (black) is shown loaded onto CD1D (light blue). Residues with nonsynonymous substitutions were mutated in silico with the lowest energy rotamer. Residues were subsequently colored based on expected interactions with CD1D (royal blue), nonsynonymous mutations with conserved biochemical properties (green), and nonsynonymous mutations with differing biochemical properties (red). **b** The TRAV10 CDR1α and TRAJ18 CDR3α gene segments that are present as a germline rearrangement in iNKT cells were examined across the genomes of ten simian primates, and sections crucial for binding are boxed. Residues important for TCR binding to CD1D and α-GalCer are shown in blue and are highly conserved. Conservative mutations are shown in green, and non-conservative substitutions in red. **c** The rhesus iNKT TCR was transduced into Jurkat cells and stained with human CD1D-α-GalCer or control tetramer. Data are expressed as percent tetramer-positive events of anti-mouse TCR-positive cells and are representative of at least three independent experiments

Next, we sought to define the molecular requirements for antigen recognition by iNKT cells in rhesus macaques. Published data suggest that the invariant TCR-α uses the same variable (TRAV10) and joining (TRAJ18) genes as the human iNKT cell TCR, but the composition of the TCR-β chain has not been determined (Kashiwase et al. 2003). We used PCR and template-switched PCR to clone the dominant TCR-α and TCR-β respectively from our rhesus-derived iNKT cell line (Table 1). We found that the TCR-α consisted of a germline rearrangement of TRAV10 and TRAJ18 with CDR3α amino acid sequence that was virtually identical to that observed in humans (Dellabona et al. 1994; Porcelli 1993). Specifically, we noted that residues known to be important for binding α-GalCer-loaded CD1D were highly conserved except for a single conservative substitution located in the CDR3α (Table 1, Fig. 3a) (Borg et al. 2007). Importantly, sequence variants located outside of the CDR3α provided confirmation that the cloned TCR was indeed derived from rhesus macaque and not from contaminating human DNA template (Table 1). These data led us to consider whether the germline-encoded gene segments constituting the invariant TCR-α were conserved across primate species. We extracted TRAV10 and TRAJ18 gene segments from the genomes of ten simian primates studied above and performed sequence alignment. We noted two clusters broadly dividing into human and Old World monkeys in one cluster and New World monkeys in the other cluster (Fig. 3b). In all species, the CDR1α and CDR3α sequences, which are known to be critical for binding α-GalCer and CD1D, were highly conserved. Together with the data presented in Fig. 1, these data suggest purifying natural selection on the genes required for lipid antigen presentation and recognition in primates.

**Table 1:** iNKT TCR-α and TCR-β gene segment usage and sequences for human and rhesus macaque with differences in italics

		Human	Rhesus Macaque
TCR-α	Variable	TRAV10*01	TRAV10*01
	CDR1	VSPFSN	VSPFSN
	CDR2	MTFSENT	MTFSENT
	CDR3	CVVSDRGSTLGRLYF	CVVSDRGSTLG*K*LYF
	Joining	TRAJ18*01	TRAJ18*01
TCR-β	Variable	TRBV25-1*01	TRBV25-1*01
	CDR1	MGHDK	MGHDK
	CDR2	SYGVNS	SYGVNS
	CDR3	CASSEEGALKESVGTQYF	CASS*DPDNEQFF*
	Joining	TRBJ2-3*01	TRBJ2-1*02

We found the dominant TCR-β rearrangement to consist of TRBV25 and TRBJ2, which is precisely what is observed in the canonical iNKT cell TCR in humans (Dellabona et al. 1994; Porcelli 1993). However, there was a conservative (K57R) (denoting the human residue, the residue number, then the rhesus macaque residue) and an aliphatic to aromatic (L60F) substitution in the CDR2β. Further, the CDR3β sequence was only 12 amino acids in length compared to 18 amino acids in humans (Table 1). We modeled the effect of these differences on the human iNKT cell TCR-CD1D-α-GalCer co-crystal structure (Fig. 3a) (Borg et al. 2007). Dynamic modeling studies indicated that this difference might influence the molecular interactions with human CD1D-α-GalCer. To directly test this hypothesis, we transduced the rhesus iNKT TCR into Jurkat cells and attempted to stain the cells with human CD1D tetramer. Only CD1D tetramer loaded with α-GalCer, and not mock-loaded tetramer, was able to successfully stain the Jurkat transductants (Fig. 3c). These data reveal that a highly conserved “pan-primate” iNKT TCR is necessary and sufficient for binding α-GalCer-loaded human CD1D tetramer.

### Non-human primate iNKT cells express diverse tissue phenotypes

**Fig. 4:**
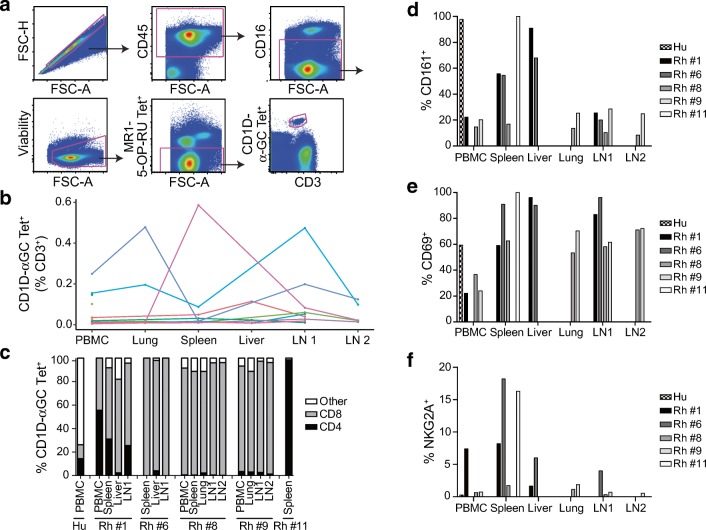
Tissue phenotypes of iNKT cells in rhesus macaques. PBMC and tissues from healthy rhesus macaques (Rh) were stained with human (Hu) α-GalCer-loaded CD1D tetramers (CD1D-ɑGC Tet). **a** Gating strategy for identifying iNKT cells from rhesus macaque blood and tissue samples proceeds from singlets to CD45^+^ cells (pan leukocytes) to CD16^−^ cells to remove NK cells. Viable cells were then identified, and MR1-5-OP-RU tetramer was used to exclude MAIT cells. Finally, CD3^+^CD1D-αGC Tet^+^ cells were identified. **b** Frequency of iNKT cells in PBMC and associated tissues from 12 rhesus macaques following staining with CD1D-αGC tetramer staining. Data are presented as the percentage of CD1D-ɑGC tetramer-positive cells among CD3^+^ cells. All tissues from the same animal are identified by color. **c** Co-receptor usage, **d** CD161 expression, **e** CD69 expression, and **f** NKG2A expression among iNKT cells from rhesus macaque PBMC and tissues identified in Online Resource 3 expressed as a percentage of all CD1D-ɑGC tetramer-positive cells

Finally, we used human CD1D tetramers to characterize the ex vivo frequencies and phenotypes of iNKT cells in peripheral blood and tissues of 12 rhesus macaques that had undergone necropsy after malaria challenge or BCG vaccination (Fig. 4a). We incorporated α-GalCer-loaded CD1D tetramers into a 20-color flow cytometry panel designed to measure co-receptor usage (CD4, CD8), memory status (CD45RA, CCR7, CD28, CD127), activation (CD69, CD161, HLA-DR, PD-1, NKG2A), and TCR-γδ usage (pan-γδ, Vγ9, Vδ1) (Online Resource 2). In seven animals, iNKT cells were rare or undetectable in both blood and tissue (Fig. 4b). We defined iNKT cell phenotypes in 17 samples derived from five animals with clearly distinguished populations of tetramer-positive cells (Online Resource 3). Most iNKT cells from these 17 samples expressed a CD45RA^−^CCR7^lo^CD28^+^ effector memory phenotype while CD127 was generally not detected (Online Resource 4a-c). Consistent with the published literature, we found that iNKT cells in the blood of rhesus macaques predominantly expressed the CD8 co-receptor in contrast to human iNKT cells (Fig. 4c) (Chen et al. 2009; Kawashima et al. 2003). Expression of CD8 was also dominant in all of the tissue samples except the spleen of rhesus #11 in which all the iNKT cells expressed CD4. Further investigation revealed that these cells were γδ T cells expressing Vγ9 and Vδ1 gene segments (Online Resource 4d-g). iNKT cells observed in spleens of three other animals were not found to contain γδ-expressing iNKT cells (Online Resource 4d). We were unable to detect iNKT cells above background in the spleens of five other animals (Online Resource 3). Next, we examined expression of CD161, which has been reported to be universally absent on blood iNKT cells in rhesus macaques but present on blood iNKT cells in humans (Exley et al. 1998; Gansuvd et al. 2003). We found that CD161 was indeed expressed by tissue-resident iNKT cells, and low staining was also observed in PBMC of some animals, challenging the current paradigm (Fig. 4d). CD69 was also more highly expressed by tissue-resident iNKT cells compared to those in blood (Fig. 4e). Finally, we examined the expression of activation marker HLA-DR and inhibitory marker PD-1, which were generally absent on iNKT cells in blood and tissue (Online Resource 4h-i). However, the inhibitory marker NKG2A was highly expressed in the spleens of three animals while being absent in the blood (Fig. 4f). Taken together, these data reveal that iNKT cells in rhesus macaques express diverse tissue phenotypes that are not always apparent in peripheral blood.

## Discussion

In summary, we performed a detailed evolutionary analysis of primate CD1 genes and found remarkable conservation across the five genes constituting the human locus, which is the best studied. We also show that molecular interactions between CD1D and the iNKT TCR are highly conserved between humans and rhesus macaques, and that this likely extends to other non-human primate species as well. Finally, we discovered diverse tissue phenotypes of iNKT cells in rhesus macaques that mirror what is observed in humans. Together, these data reveal a remarkable shared history within a specialized subset of primate T cells.

Though CD1 genes are present in all examined mammalian genomes, the number of functional CD1 genes varies from 1 to 13, depending on the species. If one were to include pseudogenes as well, this number would be even higher. In this context, with the exception of a single-gene duplication of CD1A in olive baboons, it is remarkable that the simian CD1 locus has remained static with exactly five syntenic CD1 genes. These data significantly extend the known evolutionary history of CD1 in a number of ways. First, our data support the existing consensus that CD1D is the last common ancestor for all CD1 genes in primates. CD1E was likely the second paralog to emerge by gene duplication, and upon neofunctionalization, provided a non-redundant function (antigen processing) compared to its parent CD1D (antigen presentation) (de la Salle et al. 2005). Finally, CD1B, CD1C, and CD1A duplicated most recently, and in the case of CD1A, have repeatedly experienced strong selection to diversify its antigen-binding domain (data not shown). The current paradigm of mammalian CD1 is quite anthropomorphic, with annotations based on homology to the five human genes. Thus, it is not known if there are more than these five genes in non-simian mammals or a different set of genes in other animals.

The evolution of MHC among primates as a whole appears to have followed a very different path, especially among New World monkeys (Adams and Parham 2001). Perhaps a closer analogy to the situation for CD1 described here are the non-polymorphic antigen-presenting genes within the MHC locus, such as HLA-E. HLA-E binds peptides derived from HLA-A leader sequences and acts as an inhibitory ligand for the CD94:NKG2A receptor of NK cells (Braud et al. 1998). Homologs of human HLA-E have been described in apes, Old World, and New World monkeys (Boyson et al. 1995; Knapp et al. 1998). Specifically, the peptide-binding groove is highly conserved with only three amino acid differences across all primate HLA-E sequences (O’Callaghan et al. 1998). This is quite similar to what we report for the CD1 sequences. For HLA-E, maintaining a conserved mode of interaction between NK cells and other somatic cells provides a consistent way to achieve tumor or immune surveillance. Like CD1, HLA-E has also been shown to bind a more diverse array of antigens and stimulate T cells via their T cell receptors (Hansen et al. 2016). The benefit here may be the activation of T cells with an “innate”-like phenotype to provide early protection against infectious challenge.

We discovered several tissue-specific phenotypes of iNKT cells in rhesus macaques that have not been previously appreciated. Tissues were obtained from animals undergoing necropsy after malaria challenge or BCG vaccination, so it is possible that some of these tissue phenotypes might not be present in naïve animals. In one animal, we found iNKT cells that expressed TCR-γδ, specifically the Vγ9 and Vδ1 gene segments. Exactly this phenotype has been described among blood iNKT cells in humans (Uldrich et al. 2013). The crystal structure of CD1D-α-GalCer-TCR-γδ revealed that Vδ1 dominated interactions with the iNKT TCR. Other studies have confirmed a role for Vδ1 in mediating recognition of CD1C and CD1D presented lipid antigens in humans (Bai et al. 2012; Ly et al. 2013; Porcelli et al. 1992). Our results suggest that the Vδ1 paradigm may also extend to rhesus macaques. We also discovered high levels of CD161 on iNKT cells in tissues, but not blood, of rhesus macaques. CD161 is an established marker of iNKT cells in both blood and tissues of humans but has not been reported previously in studies of rhesus macaques, most of which have focused on blood (Gansuvd et al. 2003). In humans, CD161 was recently shown to act as a co-stimulatory molecule and mark T cells with an innate-like transcriptional signature (Fergusson et al. 2014). In rhesus macaques, CD161^+^CD8^+^ T cells were found to be enriched at mucosal sites compared to blood and showed enhanced IFN-γ, IL-17, and perforin production (Rout, 2016). Thus, it is highly likely that the functional profiles and transcriptional signatures of iNKT cells in rhesus macaques mirror that of humans. One notable contrast that has been reported previously and confirmed by the data presented here is that iNKT cells in rhesus macaques are typically CD4^−^CD8^+^ while those in humans are mostly CD4^+^CD8^−^ or CD4^−^CD8^−^ (Fig. 4c) (Gansuvd et al. 2003). The role of the co-receptor in facilitating antigen recognition and function of iNKT cells has not been thoroughly investigated. One study showed that anti-CD4 specifically inhibited proliferation and cytokine secretion but not cytotoxicity by CD4^+^ human iNKT cells (Chen et al. 2007). In mice, iNKT cells are positively selected by homotypic interactions of CD4^+^CD8^+^ thymocytes and then emerge expressing only the CD4 co-receptor (Matsuda and Gapin 2005). The developmental program of iNKT cells in thymus from rhesus macaques can now also be probed using the approach described here.

We discovered that the iNKT TCR-β chain was composed of TRBV25-1, also known as Vβ11. These results apparently contradict published data suggesting iNKT cells in rhesus macaques do not express Vβ11 (Gansuvd et al. 2003; Motsinger et al. 2003). Those studies used a monoclonal antibody targeting human Vβ11, so it is possible that the binding epitope is absent in rhesus macaques. Indeed, we have observed that anti-human Vβ11 fails to stain PBMC from rhesus macaques, supporting this hypothesis (data not shown). By contrast, we defined the TCR using template-switched PCR and show that it is sufficient to bind human CD1D tetramer after lentiviral transduction of Jurkat cells. We found the CDR3β sequence to consist of 12 amino acids compared to 18 in the canonical human iNKT TCR (Borg et al. 2007). Despite this marked reduction, TCR transduction studies confirmed the ability of human CD1D-α-GalCer tetramer to bind rhesus-derived iNKT TCR. A study of 54 human iNKT TCRs revealed variability of the CDR3β sequence motif ranging from 11 to 18 amino acids, which is consistent with our finding (Chamoto et al. 2016).

Finally, we confined our molecular and functional analysis to CD1D and iNKT cells, but this approach could readily be applied to other CD1 genes. A recent study showed that rhesus CD1B molecules were able to bind and present a mycobacterial glycolipid to human CD1B-restricted T cells (Morita et al. 2008). However, GMM was presented by CD1C to CD8^+^ T cells in rhesus macaques compared to CD1B and CD4^+^ T cells in humans (Kasmar et al. 2011; Morita et al. 2013). These data suggest that the cross-species conservation in CD1D and iNKT cell TCR interactions that we note here could extend to other CD1-restricted T cells, such as CD1B-restricted germline-encoded mycolyl-reactive (GEM) T cells (Van Rhijn et al. 2013).

## Electronic supplementary material

Online Resource 1 (PDF 70 kb)

Online Resource 2 (PDF 79 kb)

Online Resource 3 (PDF 795 kb)

Online Resource 4 (PDF 585 kb)
