# Supplementary material for: Conservation of molecular and cellular phenotypes of invariant NKT cells between humans and non-human primates
Source: Immunogenetics. 2019 May 23;71(7):465–78. doi: 10.1007/s00251-019-01118-9 (PMC6647187; doi:10.1007/s00251-019-01118-9)
Supplement: Supplementary file 1 — (PDF 70 kb) [file 251_2019_1118_MOESM1_ESM.pdf]

**ID**	**Vaccination**	**Route**	**PBMC**	**Spleen**	**Liver**	**Lung**	**Lymph Nodes**
Rhesus #1	Malaria	ID	x	x	x		x
Rhesus #2	BCG	ID and AE	x				
Rhesus #3	BCG	ID and AE	x				
Rhesus #4	BCG	IV	x	x		x	x
Rhesus #5	BCG	ID and AE	x				
Rhesus #6	Malaria	IM	x	x	x		x
Rhesus #7	Malaria	IV	x	x	x		x
Rhesus #8	BCG	IV	x	x		x	x
Rhesus #9	BCG	AE	x	x		x	x
Rhesus #10	BCG	IV	x	x		x	x
Rhesus #11	BCG	AE	x	x		x	x
Rhesus #12	Malaria	IM	x	x	x		x
**12**			**12**	**9**	**4**	**5**	**9**

Online Resource 1 Tissue list and vaccination history of 12 rhesus macaques included in this study. Rhesus macaques were challenged with malaria or given BCG vaccinations as part of other studies. The route of challenge or vaccination indicated: AE = aerosol, ID = intradermal, IM = intramuscular, IV = intravenous.

Yu KKQ, Wilburn DB, Hackney JA, Darrah PA, Foulds KE, James CA, Smith MT, Jing L, Seder RA, Roederer M, Koelle DM, Swanson WJ, Seshadri C*. Conservation of molecular and cellular phenotypes of invariant NKT cells between humans and non-human primates. Immunogenetics. *Corresponding author – Department of Medicine, University of Washington, Seattle, WA USA
