# Supplementary material for: Conservation of molecular and cellular phenotypes of invariant NKT cells between humans and non-human primates
Source: Immunogenetics. 2019 May 23;71(7):465–78. doi: 10.1007/s00251-019-01118-9 (PMC6647187; doi:10.1007/s00251-019-01118-9)
Supplement: Supplementary file 2 — (PDF 79 kb) [file 251_2019_1118_MOESM2_ESM.pdf]

Specificity	Purpose	Fluorophore	Clone	Supplier
Vδ1	γδ T cells	FITC	TS8.2	ThermoFisher Scientific
CCR7	Memory	BB700	3D12	BD Biosciences
Pan-γδ TCR	γδ T cells	PE	5A6.E9	ThermoFisher Scientific
CD69	Activation	ECD	TP1.55.3	Beckman Coulter
CD28	Memory / Differentiation	PE Cy5	CD28.2	BioLegend
CD4	Lineage	PE Cy5.5	MHCD0418	ThermoFisher Scientific
CD45RA	Memory	PE Cy7	L48	BD Biosciences
NKG2a	NK cells	APC	Z199	Beckman Coulter
Vγ9	γδ T cells	Ax680	7A5	ThermoFisher Scientific
CD3	Lineage	APC Cy7	SP34-2	BD Biosciences
CD8	Lineage	BV395	RPA-T8	BD Biosciences
Dead Cell Stain	Viability	UV dye		ThermoFisher Scientific
CD16	NK cells	BUV496	3G8	BD Biosciences
HLA-DR	Activation	BUV661	G46-6	BD Biosciences
CD127	Memory	BUV737	HIL-7R-M21	BD Biosciences
MR1-5-OP-RU Tet	MAIT cells	BV421		NTCF
CD45	Pan-leukocyte	BV510	D058-1283	BD Biosciences
CD161	MAIT cells	BV605	HP-3G10	BioLegend
CD1D-PBS-57 (α-GalCer) Tet	iNKT cells	BV711		NTCF
PD-1	Exhaustion	BV785	EH12.2H7	BioLegend

Online Resource 2 The results reported in this manuscript that explored the frequencies of α-GalCer -specific T cells in rhesus macaque tissues were obtained using a 20-color flow cytometry panel. In addition to the α-GalCer tetramer discussed in the manuscript, an additional tetramer, MR1-5-OP-RU (Corbett et al., 2014), was included for identification of mucosal associated invariant T (MAIT) cells, an innate-like T cell subset that express an invariant TCR-α chain and a diverse TCR-β chain. MAIT cells recognize vitamin B metabolites that are presented by the antigen-presenting molecule, MR1. The tetramers were obtained from the NIH Tetramer Core Facility (NTCF). Additional markers in the panel allowed us to identify γδ T cells, distinguish between T cell lineages, and determine the memory phenotypes and activation statuses of each T cell population.

Yu KKQ, Wilburn DB, Hackney JA, Darrah PA, Foulds KE, James CA, Smith MT, Jing L, Seder RA, Roederer M, Koelle DM, Swanson WJ, Seshadri C*. Conservation of molecular and cellular phenotypes of invariant NKT cells between humans and non-human primates. Immunogenetics. *Corresponding author – Department of Medicine, University of Washington, Seattle, WA USA
