# Supplementary material for: Conservation of molecular and cellular phenotypes of invariant NKT cells between humans and non-human primates
Source: Immunogenetics. 2019 May 23;71(7):465–78. doi: 10.1007/s00251-019-01118-9 (PMC6647187; doi:10.1007/s00251-019-01118-9)
Supplement: Supplementary file 3 — (PDF 795 kb) [file 251_2019_1118_MOESM3_ESM.pdf]

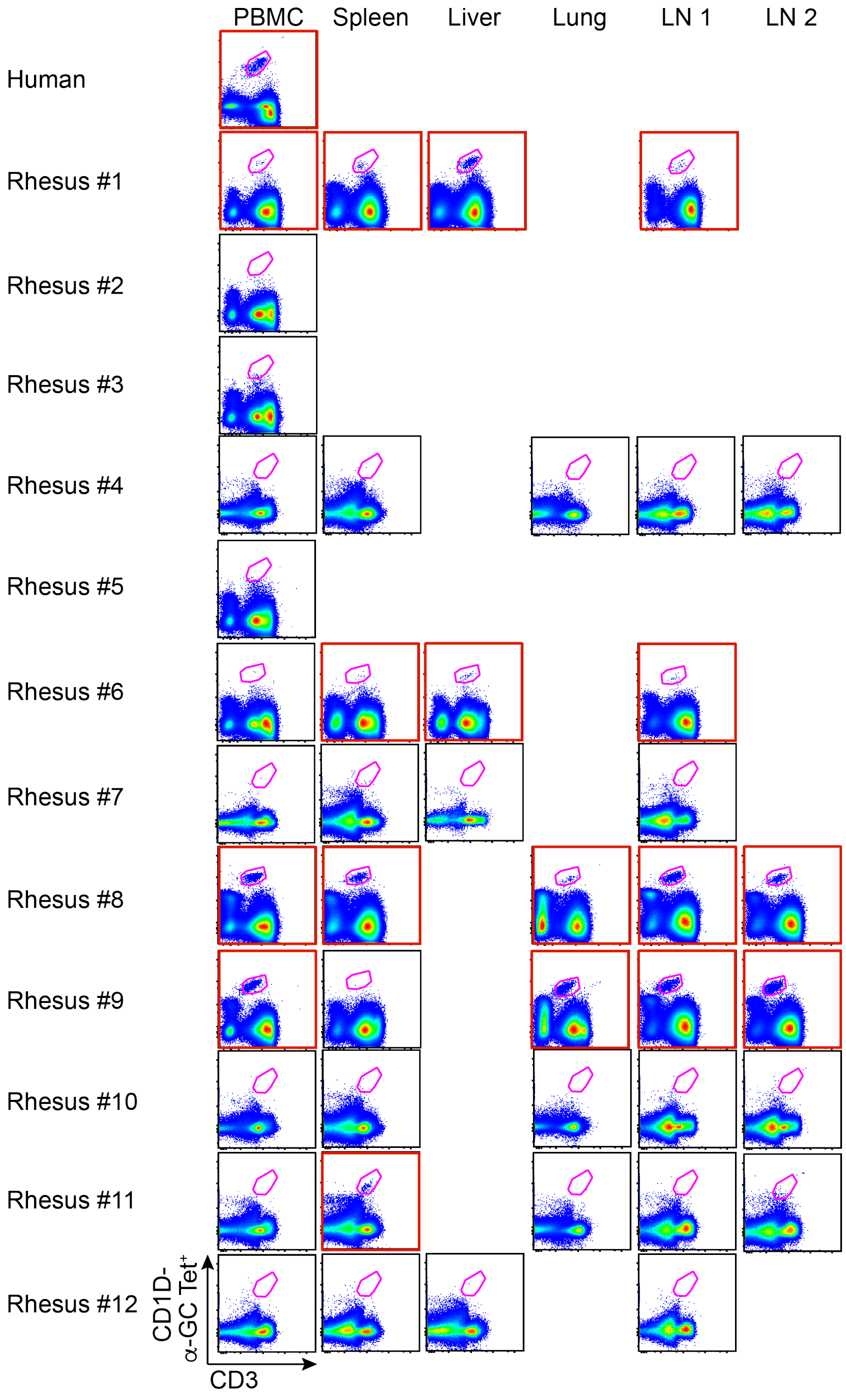

Online Resource 3 Tissues from 12 rhesus macaques were analyzed for the presence of iNKT cells using human CD1D-α-GalCer tetramer. Two lymph node (LN) samples were obtained from each of five animals. Human PBMC were also stained as a positive control. Of the 44 samples analyzed, iNKT cells were clearly observed as distinct populations in 17 samples (indicated with red boxes). These 17 samples were further analyzed for expression of surface markers as reported in the manuscript.

Yu KKQ, Wilburn DB, Hackney JA, Darrah PA, Foulds KE, James CA, Smith MT, Jing L, Seder RA, Roederer M, Koelle DM, Swanson WJ, Seshadri C*. Conservation of molecular and cellular phenotypes of invariant NKT cells between humans and non-human primates. Immunogenetics. *Corresponding author – Department of Medicine, University of Washington, Seattle, WA USA
