# Supplementary material for: Conservation of molecular and cellular phenotypes of invariant NKT cells between humans and non-human primates
Source: Immunogenetics. 2019 May 23;71(7):465–78. doi: 10.1007/s00251-019-01118-9 (PMC6647187; doi:10.1007/s00251-019-01118-9)
Supplement: Supplementary file 4 — (PDF 585 kb) [file 251_2019_1118_MOESM4_ESM.pdf]

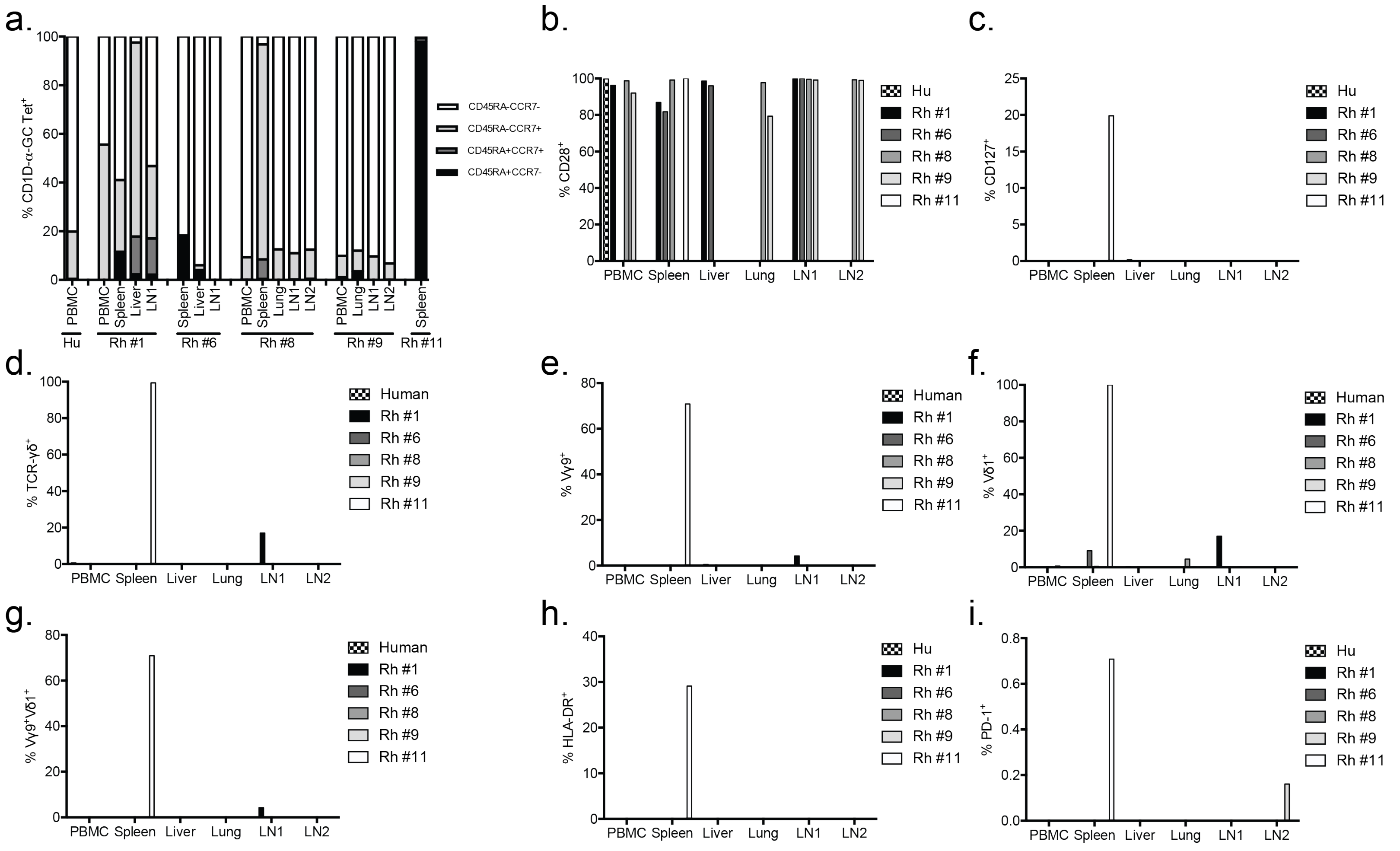

Online Resource 4 Comparative analyses of iNKT cells found in 17 tissue samples derived from five rhesus macaques (Rh) compared with human (Hu) PBMC. Surface markers that were tested include those for co-receptor (CD4, CD8), memory (CD45RA, CCR7, CD28, CD127), activation (CD69, CD161, HLA-DR, PD-1, NKG2A), and TCR expression (γδ, Vγ9, Vδ1). Tissues examined include PBMC, spleen, liver, lung, and lymph nodes (LN).

Yu KKQ, Wilburn DB, Hackney JA, Darrah PA, Foulds KE, James CA, Smith MT, Jing L, Seder RA, Roederer M, Koelle DM, Swanson WJ, Seshadri C*. Conservation of molecular and cellular phenotypes of invariant NKT cells between humans and non-human primates. Immunogenetics. *Corresponding author – Department of Medicine, University of Washington, Seattle, WA USA
